# Lower Limb Core Scale: A New Application to Evaluate and Compare the Outcomes of Bone and Soft-Tissue Tumours Resection and Reconstruction

**DOI:** 10.1155/2014/652141

**Published:** 2014-08-03

**Authors:** Andrea Monticelli, Davide Ciclamini, Michele Boffano, Elena Boux, Paolo Titolo, Bernardino Panero, Bruno Battiston, Raimondo Piana, Pierluigi Tos

**Affiliations:** ^1^U.O.C Muscoloskeletal Traumatology, U.O.D. Microsurgery, AO Città Della Salute e Della Scienza, C.T.O. Hospital, via Zuretti 29, 10100 Torino, Italy; ^2^U.O.C Reconstructive and Oncologic Orthopaedics, AO Città Della Salute e Della Scienza, C.T.O. Hospital, Torino, Italy

## Abstract

Several methods are used to evaluate the functional outcome of tumour resections and reconstructions in the lower limb. However, one of their most common limitations is that they are specifically developed to evaluate only oncological patients. We introduced the Lower Limb Core Scale (LLCS) to overcome this limitation. The aim of this study was to evaluate the functional and subjective results in the lower limb and to evaluate the use of the LLCS. We conducted a retrospective cohort study using various tools to investigate the outcomes. The results of the LLCS were correlated with the results of other functional tests. A total of 44 patients were included in the study. None of the demographic variables correlated with the functional or health-related quality of life (QoL) scores except for gender, whereby male patients had an increased functional score. The correlation between LLCS and other scores was positive (*r* ^2^ = 0.77). The satisfactory QoL scores, and functional outcomes scores indicated the LLCS to be a reliable option for general and specific evaluation of lower limb reconstructions. We suggest using the LLCS for comparisons of oncological reconstructions with lower limb reconstructions in different disciplines.

## 1. Introduction

Bone and soft-tissue tumours of the limbs represent a group of differing and rare diseases that in the past normally required debilitating surgical operations. In the mid-1970s, the rate of amputation for extremity soft-tissue sarcomas was 40–50% [1]. Since then, the combination of surgery and radiotherapy has been proved to yield superior local control of tumours, compared with local excision alone, and has been fundamental to the adoption of limb-sparing surgery for high-risk soft-tissue sarcomas of the extremities [2, 3]. The current multimodal approaches, combining wide surgical resection with radiotherapy and/or chemotherapy, allow limb preservation in 90–95% of patients [4–6]. In fact, limb salvage is considered the cornerstone of treatment for musculoskeletal sarcoma of the extremities if a functional limb can be attained and no oncological contraindications are present [6]. Surgical margins are the most important factor associated with local tumour control [7], but obtaining good oncological margins can also result in extensive or critical loss of bone and soft-tissue components [8, 9].

Pedicled and free tissue transfers have been popularised for limb preservation and are useful techniques for bone and soft-tissue reconstruction [10, 11], since the principal objective, after local and distant control of the disease, must remain the residual functionality (functional outcome) and the quality of life (QoL) of the patient.

Several methods are used to evaluate the functional outcome in lower limb disease. However, they have several limitations, one of the most common being that they are specifically developed to evaluate only oncological patients. Considering the low prevalence of these diseases, such constraint limits the knowledge about long-term outcomes of reconstructive surgery, especially in cases where comparison of different groups of patients with different medical conditions is required. For this reason we have introduced the Lower Limb Core Scale (LLCS), a simple and rapid tool that is not disease specific. LLCS is a global scale developed by the American Academy of Orthopaedic Surgeons (AAOS) in order to form a scale that had acceptable face validity for all musculoskeletal specialists and could be used to assess all lower limb problems. See appendix for detailed information about LLCS. The LLCS may be assigned either to a lower limb as a whole or to a specific joint or side without sacrificing its reliability, which is a useful asset given the variability of reconstructive procedures. It is already successfully used in hip prosthetic surgery [12] combined with SF-12, where it showed that high-grade acetabular defects in revision prosthetic surgery can be fixed without bone graft, using jumbo cups and obtaining a good functional and QoL result. It also has been used for femoral lengthening surgery [13] combined with SF-36, pre- and postoperatively in order to compare 2 different surgical techniques: femoral lengthening over a nail and internal lengthening nails. It appeared to be useful because it demonstrated the improvement in functionality postoperatively, without significative differences between the two techniques. It is not used in oncologic orthopaedics yet.

The aim of this study was to evaluate, using LLCS, the functional and subjective results of 44 resections of bone and soft-tissue sarcomas of the lower limb and the reconstruction with microsurgical free or local flaps. We propose the LLCS as a new means to evaluate the outcomes of reconstructive microsurgery in lower limb sarcomas and compare it herein with already available tools.

## 2. Materials and Methods

This was a retrospective cohort study. The records of 92 patients who had undergone lower limb reconstructive surgical therapy for bone and soft-tissue tumours in our department during the period 1998–2013 were reviewed and analysed. All patients were treated by the same multidisciplinary team consisting of oncological surgeons (resection of the tumour) and microsurgeons (reconstruction). Free informed consent to participate in the study was obtained from all patients. The evaluation tools were administered during the follow-up visit of the patient. The order in which these tools were administered was kept consistent for each patient, and in the same order presented here.

The diagnostic criteria for the inclusion of patients were based on histological and clinical findings. The study included patients with benign and malignant tumours of the lower limb in need of extensive resection of bone and soft tissue as well as reconstruction. The inclusion criterion for the reconstructive procedure was soft tissue, bone, or combined major resection and subsequent coverage with local or free flap. Furthermore, to be included patients had to (1) have not undergone surgery following amputation; (2) be at least 15 years of age; (3) have no evidence of progression of disease; and (4) be reachable and compliant with the study.

The basic principles of the microsurgical approach were to undertake immediate primary reconstruction so as to ensure resection with adequate clear margins, avoid limb amputations, and achieve immediate adequate coverage of the tissues. In cases requiring complex reconstructions, the approach involved working in two teams simultaneously.

Three tools were used to investigate the quality of life (QoL): (1) the Eastern Cooperative Oncology Group test (ECOG); (2) the EuroQol 5-dimension and the EuroQol Visual Analogue Scale (EQ-5D and EQ-VAS); and (3) the Short Form-36 version 2 (SF-36v.2). Three tools were used to investigate the functional outcome: (1) the Musculoskeletal Tumor Society Scale (MSTS); (2) the Toronto Extremity Salvage Score (TESS); and (3) the Lower Limb Core Scale (LLCS).

### 2.1. ECOG

The ECOG scales and criteria are useful to assess how a patient's disease is progressing and how the disease affects the daily living abilities of the patient and to determine appropriate treatment and prognosis. This test is commonly administered by the physician [14].

### 2.2. EuroQol

EuroQol measures specific health status. It consists of (1) five questions on mobility, self-care, usual activity, pain/discomfort, and anxiety/depression and (2) a 20 cm vertical visual analogue scale (E-VAS). It is a self-administered scale for evaluation of QoL [15].

### 2.3. SF-36v.2

This questionnaire measures health-related quality of life (HRQoL) and is self-administered [16].

### 2.4. MSTS

This test is self-administered and is used to identify the participants' subjective functional abilities.

### 2.5. TESS Lower-Extremity Version

This self-administered questionnaire allows participants to indicate the level of difficulty experienced in dressing, grooming, mobility, work, sports, and leisure [17].

### 2.6. LLCS

See the Appendix for details [18].

### 2.7. Statistical Analysis

Means, median, and standard deviations for all variables were calculated. The association between the independent variables (gender, age > 55 years, disease site, expression of the disease, incidence of recurrence, incidence of complications, and type of limb) was assessed by the odds ratio (OR) considering statistically significative a result with a *P* < 0.05. We correlated the LLCS results with the mean results of the other functional tests. The correlation coefficient (*r* ^2^) was used to measure the strength and direction of a linear relationship between two quantitative variables. This value is between −1 and 1, where −1 indicates the maximum negative correlation and 1 indicates the best positive correlation.

## 3. Results

A total of 44 (22 were female and 22 were male) patients included in the study were visited and underwent a functionality and QoL evaluation. Patients had an age range of 16–93 years (mean: 54 years). Forty-eight patients who did not meet the eligibility criteria were excluded. Seven patients were excluded because they subsequently underwent amputation, 21 were excluded because they were deceased at the time of the study, and 1 patient was excluded because he did not match the age criteria. 19 patients were excluded because they were not reachable.

All 44 patients showed no evidence of disease. See the summary table (Table 1). Fourteen patients showed early or later postoperative complications, 12 of whom were potentially related to surgery and two of whom were not surgical-related (Table 2).

Several flaps were used (Table 3). Patients were finally divided into two different groups based on the type of limb and the reconstruction performed:

- Group 1: “functional limb” (limbs reconstructed to restore a muscular or bone function); eight patients were listed in this group (Table 3).
- Group 2: “coverage limb”; 36 patients were listed in this group (Table 3).

The correlations of each of the demographic and clinical variables with the functional and HRQoL measures are listed in Tables 4, 5, and 6. The range of ECOG is 0–5, where 0 is the normal QoL and 5 is the death of the patient. The range of EQ-5D is 0-1, where 1 is the normal QoL and 0 is the total inability of the patient. The range of EQ-VAS is 0–100, where 100 is the normal QoL and 0 is the worst QoL. The range of MSTS is 0–100, where 100 is the normal functionality and 0 is the total inability of the patient. The range of TESS is 0–100, where 100 is the normal functionality and 0 is the total inability of the patient. The range of SF-36, for all the raw scores, is 0–100. The ranges of the global normalized scores, physical component summary (PCS) and mental component summary (MCS), are approximately, respectively, as follows: 17–57 for the PCS and 17–62 for the MCS.

None of the demographic variables correlated with the functional or HRQoL scores except for gender, whereby male patients had an increased functional score (LLCS: OR 12, *P* = 0.03; TESS: OR 13, *P* = 0.01). The other clinical and demographic variables had no correlations with the functional and HRQoL scores.

**Figure 1:**
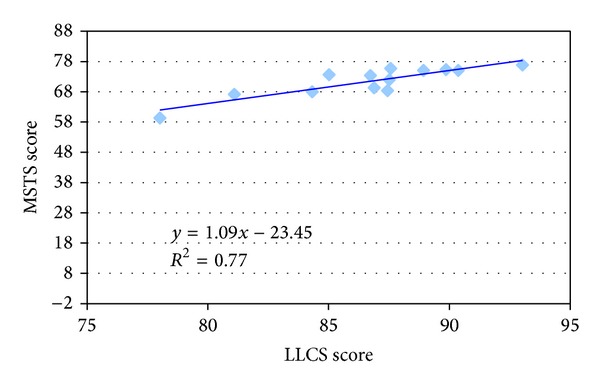
Correlation between MSTS score (range: 0–100) and LLCS score (range: 0–100).

**Figure 2:**
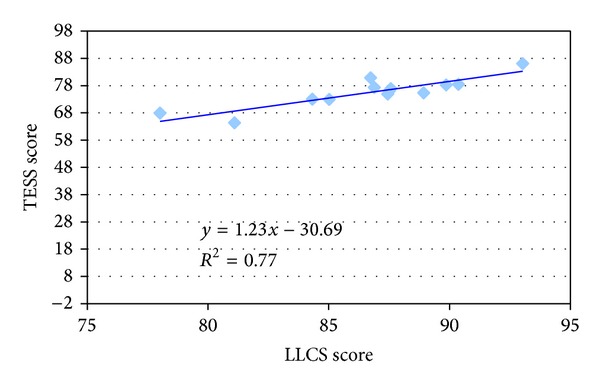
Correlation between TESS score (range: 0–100) and LLCS score (range: 0–100).

The correlation between LLCS and MSTS and LLCS and TESS showed in both cases an *r* ^2^ of 0.77 (Figures 1 and 2).

## 4. Discussion

Radical resection and limb-salvage surgery are currently recommended as the treatment of choice for sarcomas in resectable soft-tissue and bone sarcomas and tumours of the extremities [19]. However, this cannot be achieved without an adequate reconstructive procedure, including sometimes pedicled or free tissue transfer [20, 21]. Recent advances in the microsurgical techniques and the associated technologies, coupled with a better understanding of microvascular anatomy, have enabled surgeons to carry out single-stage reconstruction covering wide and composite tissue losses in any anatomical location. The aim of this study was to evaluate, with the aid of LLCS, the functional and subjective results in our patients and to propose a new application of the LLCS as a new means to evaluate the outcomes of flap reconstruction in lower limb bone and soft-tissue tumours and sarcomas, in addition to the already available tools.

Our comparison of the MSTS (Enneking score) [22] and TESS with the LLCS, as measures of postoperative outcome for patients with tumours of the lower limb, showed high and moderate correlations of most LLCS scores regardless of the location of the tumour (Table 3). Despite being a new tool, the LLCS score has been shown to be a useful measure for evaluating the physical disability of patients with pathological conditions not in a fixed anatomical location, such as tumours.

Our study did not find significant differences between the general lower limb functions of the patients who had limb-sparing leg surgery and those with limb-sparing thigh surgery, which would support the assertion that if patients who have undergone limb-salvage surgery are considered to be a single cohort it is not necessarily detrimental or essential to do a comparative analysis with other surgical approaches. The correlation with the well-established scores supports the validity of the LLCS in oncological orthopaedics. Also, it is faster to administer and easy to calculate and it has been made in order to be used alone or complementary to SF-36, which is the most used QoL score. Furthermore, the LLCS could be used to compare oncology patients who have undergone complex lower limb reconstruction with other patients from nononcological specialties who have experienced similarly complex reconstructions. This aspect can be useful in order to understand if flap reconstructions can really improve the health of oncological patients comparing their results to bigger and well-studied cohorts. Data from trauma surgery of the lower limb suggest that sometimes amputation can achieve better results than high complexity reconstructions, in terms of QoL and functionality. With the LLCS we could use a common tool to evaluate and plan a better reconstruction procedure in terms of functionality, QoL, and healing time in elective surgery patients.

Our functional results are comparable to those in the literature. The only difference regarding clinical and demographic aspects is the one between male and female patients, probably related to a statistical coincidence. All of the other considered aspects showed no statistically significant differences.

Clinical and demographic factors play an important role in measuring the effect of disability on HRQoL. The SF-36 score has been validated in patients with musculoskeletal complaints and is used widely for measuring health outcomes. However, it is a generic questionnaire and has the potential disadvantage of being less sensitive to clinical change in patients with complaints specific to an anatomical region or disease process [11, 13].

This study used three different generic tests to evaluate HRQoL. The differences in HRQoL between the groups of patients were less strong than those of functional outcomes. The lack of a relationship between impairments and HRQoL found in this study was also reported in a recent paper by Marchese et al. [23], where the MSTS was not found to be correlated with HRQoL in survivors of paediatric osteosarcoma. Similarly, in a study on HRQoL in patients with spinal cord injury, the relationships between HRQoL and impairments and activity limitations were weak and inconsistent, although there was a strong and consistent relationship between restriction in participation of social activities and HRQoL [24].

Rehabilitation of these patients must focus on improving patients' perception of HRQoL not only by reducing their impairments and daily activity limitations but also by helping them reintegrate into normal life. Impairments and activity limitations are widely used to evaluate the functional disabilities of sarcoma patients. Restriction in the participation in social activities is an important aspect of functional well-being that is not always measured. QoL restrictions should be included in the functional assessment along with the other functional measures for a complete understanding of patient outcomes.

Finally, we advocate continuing validation of the LLCS in further studies in both Italian and other languages. The LLCS permits inclusion of all tumours of the lower limb of different grade, histology, and anatomical location in a single sample. We attempted to do so in an effort to allow generalization of the results to a wide variety of tumours and treatments of the lower limb. The major limitation of our study is that the LLCS in Italian is not yet validated. Also the small numbers in this series is a major limitation. Another potential limitation was our inclusion of more than one type of tumour and anatomical location of the lower limb, for reasons of generalization. The LLCS score has been shown, for the first time in flap reconstruction surgery after major lower limb tumour resections, to be a useful measure for evaluating physical disability in this population of patients. This concept strengthens our opinion that the LLCS can be used for the entire lower limb in pathology of uncertain anatomical location, such as tumours. We propose that this questionnaire be used to measure the functional status of patients who have undergone flap reconstruction (microsurgical and pedicled flaps) for sarcoma of the lower limb, although further evidence will be needed to confirm our findings.

## Figures and Tables

**Table 1:** Summary table.

Number	Patient	Gender	Age	Location	Diagnosis	Flap type
1	SR	F	80	Anterolateral proximal leg	Myxofibrosarcoma middle-high grade	Gastrocnemius muscle
2	CF	M	63	Knee	Myxofibrosarcoma	Gastrocnemius muscle
3	PG	F	44	Diaphysis tibia	Adamantinoma	Gastrocnemius muscle
4	TS	M	25	Proximal tibia	Osteoblastic osteosarcoma	Gastrocnemius muscle
5	CA	M	18	Proximal tibia	Telangiectatic osteosarcoma	Gastrocnemius muscle
6	ML	M	50	Diaphysis tibia	Synovial sarcoma	Gastrocnemius muscle
7	TV	F	73	Knee	Leiomyosarcoma	Gastrocnemius muscle
8	SA	F	67	Knee	Leiomyosarcoma	Gastrocnemius muscle
9	FH	M	16	Proximal tibia	Ewing's sarcoma	Gastrocnemius muscle
10	PB	M	52	Anterolateral proximal leg	Leiomyosarcoma	Gastrocnemius muscle
11	CC	F	93	Proximal leg	Myxofibrosarcoma	Gastrocnemius muscle
12	DF	M	21	Distal femur	Telangiectatic chondroblastic osteosarcoma	Latissimus dorsi
13	DA	F	44	Gluteal region	High-grade pleomorphic sarcoma	Latissimus dorsi
14	TA	M	62	Posterior thigh	High-grade sarcoma	Latissimus dorsi
15	RD	F	39	Diaphysis tibia	Polyostotic fibrous dysplasia	Fibula
16	LD	F	53	Proximal femur	Aneurysmatic bone cyst	Fibula
17	PM	M	39	Proximal femur	Aneurysmatic bone cyst	Fibula
18	PP	F	47	Foot tarsus	Synovial sarcoma	Free Rib
19	AA	M	53	Anterolateral proximal leg	Extra-abdominal desmoid	Parascapular
20	RR	M	41	Proximal leg	Extra-abdominal desmoid	Parascapular
21	ZG	M	71	Anterior distal thigh	High-grade leiomyosarcoma	Propeller
22	GP	F	44	Lateral thigh	High-grade myxofibrosarcoma	Propeller
23	CF	M	67	Foot tarsus	Squamous-cell carcinoma	Gracilis
24	BA	M	77	Anterolateral proximal leg	High-grade leiomyosarcoma	Sural
25	AG	F	16	Proximal tibia	Ewing's sarcoma	Axial/rotational pedicled flaps
26	BG	F	69	Sacral bone	Sacral chordoma	Gluteal
27	BM	M	81	Thigh	High-grade myxoid liposarcoma	Axial/rotational pedicled flaps
28	BV	M	55	Gluteal region	Myxofibrosarcoma	Axial/rotational pedicled flaps
29	RP	M	83	Diaphysis tibia	Myxofibrosarcoma	Axial/rotational pedicled flaps
30	PR	F	70	Medial thigh	Lipoma-like liposarcoma	Axial/rotational pedicled flaps
31	FL	F	76	Gluteal region	Hemangiopericytoma	Axial/rotational pedicled flaps
32	YE	F	81	Anterior leg	Squamous cell carcinoma	Axial/rotational pedicled flaps
33	NC	M	47	Anterolateral proximal leg	High-grade leiomyosarcoma	Axial/rotational pedicled flaps
34	AD	M	38	Knee	Myxoid liposarcoma	Axial/rotational pedicled flaps
35	IM	F	75	Proximal posterior leg	High-grade sarcoma	Axial/rotational pedicled flaps
36	AC	F	82	Gluteal region	Liposarcoma	Axial/rotational pedicled flaps
37	GI	F	28	Proximal femur	High-grade chondrosarcoma	Axial/rotational pedicled flaps
38	GV	F	63	Proximal thigh	Leiomyosarcoma	Axial/rotational pedicled flaps
39	ME	M	54	Knee	GCT (giant-cell tumor)	Axial/rotational pedicled flaps
40	BJ	F	49	Knee	High-grade liposarcoma	Axial/rotational pedicled flaps
41	BF	M	75	Proximal posterior thigh	Myxoid liposarcoma	Axial/rotational pedicled flaps
42	PE	F	18	Anterolateral proximal leg	Ewing's sarcoma	Axial/rotational pedicled flaps
43	BE	F	36	Knee	Malignant fibrous histiocytoma	Axial/rotational pedicled flaps
44	MG	M	49	Gluteal region	Fibrosarcoma	Axial/rotational pedicled flaps

**Table 2:** Number and type of complications.

Type of complication	Number
Potentially surgical-related complication	
Flap failure (partial or total necrosis)	4
Local infection	2
Nerve dysfunction	2
Pseudoarthrosis	1
Other	3

Not surgical-related complication	
TVP	1
Pneumonia	1

**Table 3:** Flap type in each group. Group 1: “functional limb”; Group 2: “coverage limb.”

Flap	Group 1	Group 2
Latissimus dorsi	2	1
Parascapular	1	1
Fibula	3	—
Propeller	—	2
Gastrocnemius	1	10
Sural	—	1
Vascularized rib	1	—
Gluteal	—	1
Gracilis	—	1
Axial/rotational pedicled flaps	—	19

**Table 4:** Scores of tests for functionality (MSTS score range: 0–100; TESS score range: 0–100; LLCS score range: 0–100).

Test	MSTS score	TESS score	LLCS scale
Total	71,85 ± 6,47	76,01 ± 7,31	87,52 ± 5,25
Males	76,82 ± 9,05	86,08 ± 3,76	93,03 ± 3,08
Females	67,1 ± 8,99	64,26 ± 8,63	81,1 ± 9,8
Age < 55	75,28 ± 4,89	78,26 ± 5,5	89,87 ± 3,65
Age > 55	67,94 ± 7,87	72,94 ± 9,4	84,33 ± 6,88
Proximal site	68,26 ± 10,15	74,86 ± 8,56	87,45 ± 10,4
Distal site	75,61 ± 7,84	76,85 ± 7,59	87,58 ± 5,36
Malignant lesion	75 ± 7,03	78,47 ± 7,12	90,38 ± 4,17
Benign lesion	59,26 ± 13,85	67,83 ± 21,15	78,02 ± 16,93
Recurrence	73,33 ± 15,04	80,84 ± 12,32	86,74 ± 9,68
Compliance	73,57 ± 13,07	72,91 ± 9,45	85,03 ± 8,87
Group 1	75 ± 11,52	75,23 ± 11,08	88,93 ± 5,54
Group 2	69,28 ± 7,73	77,3 ± 9,2	86,89 ± 6,86

**Table 5:** Scores of the tests for QoL. PCS: physical component summary; MCS: mental component summary (ECOG score range: 0–5; EQ-5D score range: 0-1; EQ-VAS score range: 0–100; SF-36 PCS and SF-36 PCM mean normalized score: 49).

Test	ECOG	EQ-5D	EQ-VAS	SF-36 PCS	SF-36 MCS
Total	0,98 ± 0,27	0,73 ± 0,08	74,42 ± 6,61	41,99 ± 4,3	50,48 ± 4,47
Males	0,77 ± 0,39	0,82 ± 0,1	80,59 ± 6,05	45,57 ± 5,2	52,81 ± 4,6
Females	1,17 ± 0,38	0,64 ± 0,13	68,52 ± 11,19	37,81 ± 6,44	47,78 ± 8,04
Age < 55	0,75 ± 0,18	0,8 ± 0,06	76,46 ± 5,47	42,33 ± 4,5	52,16 ± 3,67
Age > 55	1,24 ± 0,34	0,65 ± 0,11	72,1 ± 7,81	41,52 ± 4,23	48,2 ± 5,45
Proximal site	1,22 ± 0,46	0,68 ± 0,13	73,91 ± 8,06	41,11 ± 6,59	47,41 ± 6,51
Distal site	0,73 ± 0,26	0,77 ± 0,1	74,95 ± 10,78	42,63 ± 5,85	52,74 ± 6,04
Malignant lesion	0,86 ± 0,28	0,76 ± 0,09	75,14 ± 7,92	43,99 ± 4,54	50,81 ± 4,73
Benign lesion	1,44 ± 0,74	0,58 ± 0,23	71,56 ± 9,97	35,33 ± 9,75	49,4 ± 12,21
Recurrence	0,8 ± 0,39	0,82 ± 0,15	79 ± 17,27	42,44 ± 9,74	53,22 ± 8,47
Compliance	0,93 ± 0,52	0,71 ± 0,17	81,14 ± 9,65	41,07 ± 6,71	54,95 ± 4,96
Group 1	0,88 ± 0,44	0,77 ± 0,11	78,13 ± 11,41	39,11 ± 6,79	52,23 ± 9,36
Group 2	1 ± 0,32	0,71 ± 0,1	73,62 ± 7,69	43,39 ± 5,16	49,71 ± 4,83

**Table 6:** Completed results of SF-36. Summary of measures: PF: physical functioning; RP: role limitation due to physical problems; BP: bodily pain; GH: general health; VT: vitality; SF: social functioning; RE: role limitation due to emotional problems; MH: mental health (score range: 0–100).

SF-36	PF	RP	BP	GH	VT	SF	RE	MH
Total	64,42 ± 9,04	51,92 ± 15,6	64,04 ± 11,78	64,04 ± 8,8	62,5 ± 9,02	79,81 ± 9,99	66,67 ± 14,94	71,38 ± 7,47
Males	72,86 ± 11,46	64,29 ± 24	75,43 ± 15,26	67,71 ± 9,47	67,14 ± 10,39	85,71 ± 11,05	78,57 ± 11,02	76,29 ± 8,67
Females	54,58 ± 13,57	37,5 ± 19,54	50,75 ± 17,12	59,75 ± 16,23	57,08 ± 15,93	72,92 ± 17,8	52,78 ± 23,39	65,67 ± 12,82
Age < 55	69 ± 21,3	53,33 ± 21,3	65,87 ± 17,96	62,87 ± 17,96	63,67 ± 10,93	83,33 ± 10,58	75,56 ± 16,67	73,33 ± 9,32
Age > 55	58,18 ± 15,14	50 ± 26,08	61,55 ± 17,19	65,64 ± 13,76	60,91 ± 16,49	75 ± 19,24	54,55 ± 27,03	68,73 ± 13,27
Proximal site	60 ± 13,86	47,73 ± 23,2	61,55 ± 16,84	58,64 ± 15,25	61,36 ± 13,21	75 ± 16,13	51,51 ± 24,4	67,27 ± 9,32
Distal site	67,67 ± 13,05	55 ± 23,43	65,87 ± 18,2	68 ± 10,99	63,33 ± 13,76	83,33 ± 13,8	77,78 ± 18,41	74,4 ± 12,09
Malignant lesion	66,43 ± 7,81	54,76 ± 15,68	68,81 ± 11,96	67,52 ± 7,3	66,43 ± 8,64	83,33 ± 8,64	68,25 ± 14,88	74,1 ± 7,06
Benign lesion	56 ± 20,24	40 ± 23,67	44 ± 11,41	49,4 ± 19,1	46 ± 12,56	65 ± 20,86	60 ± 24,59	60 ± 12,29
Recurrence	69 ± 14,34	60 ± 23,67	55,8 ± 18,84	71,8 ± 7,03	62 ± 10,7	87,5 ± 10	73,32 ± 20,67	76,8 ± 10,05
Compliance	64,64 ± 14,23	48,21 ± 22,51	62,64 ± 18,82	68,23 ± 13,72	71,79 ± 12,03	88,39 ± 13,44	71,43 ± 19,38	77,43 ± 8,72
Group 1	66,88 ± 9,31	50 ± 15,95	51,25 ± 12,88	64,38 ± 11,66	78,13 ± 12,39	78,13 ± 12,39	75 ± 21,97	73 ± 12,62
Group 2	63,33 ± 14,94	52,78 ± 24,69	67 ± 18,1	69,72 ± 11,95	61,67 ± 14,23	80,56 ± 15,95	62,96 ± 22,31	70,67 ± 10,56

## Appendix

### LLCS

The LLCS is a global scale combining seven items addressing pain, stiffness, swelling, and function, performed at an acceptable level. It is included in the lower limb instruments collection and was validated in 2004 [20]. Value score range is 0–35 (raw score) or 0–100 (standardized mean) where “0” represents a poor outcome and “100” represents the best possible outcome.

The American Academy of Orthopaedic Surgeons (AAOS) developed in the 1990s a series of questionnaires in order to measure and analyze musculoskeletal outcomes from patients of all ages. Groups of clinicians and health-services researchers were asked to focus on the patient-oriented outcomes that were realistically expected to be affected by medical and surgical interventions. The agreed-on items were then tested for validity, reliability, and sensitivity. The Short Form-36 (SF-36) was selected as a companion general health status questionnaire. The Lower Limb Core Scale is a global scale that was created by combining seven items into three subscales (pain attributed to the lower limb, stiffness and swelling, and function). At the beginning the intent was to form a single core scale that had acceptable face validity for all musculoskeletal specialists and could be used efficiently to assess all lower limb problems. At the end of the first iteration of item generation, the large number of items selected suggested that the core would need to be supplemented with special items to provide flexibility and alleviate respondent burden. The preliminary individual scales had internal reliability (Cronbach's alpha) of 0.81 to 0.95.

Principal factor analyses indicated that there was considerable overlap between the new scale and the physical subscales of the SF-36. In the interest of reducing respondent burden and the time for the administration of the test, the Lower Limb Core Scale was reduced to seven items addressing pain, stiffness and swelling, and function.

The LLCS instrument combines features of existent, and in many cases substantially longer, instruments into shorter more user-friendly questionnaire. It may be used alone or supplemented by the SF-36, a widely used general health status questionnaire, because it is complementary to the SF-36. Construct validity was determined by examining patterns of correlations between it and physician and patient assessments and previously validated scales (the SF-36) and by assessing the ability of the new scale to discriminate between groups, as demonstrated by the *F* and *t*-tests.

The LLCS was found to contribute independently to prediction of the transition score constructed from the patient and physician assessments of change. It can be used with attribution of pain either to the lower limb or to a specific joint or side without sacrificing reliability.

The lower limb instruments, scoring algorithms, and description of normative data can be accessed at clicking on “Research & Quality” and clicking on “Outcomes Instruments and Information,” then on “Outcomes Instruments,” and then on “Lower Limb.”

*Lower Limb Core Scale Questionnaire*

*Instructions*. Please answer the following questions for the lower limb being treated or followed up. If it is BOTH lower limbs, please answer the questions for your worse side. All questions are about how you have felt, on average, during the past week. If you are being treated for an injury that happened less than one week ago, please answer for the period since your injury.

1. During the past week, how stiff was your lower limb? (Circle one response.)
  (1) Not at all
  (2) Mildly
  (3) Moderately
  (4) Very
  (5) Extremely.
2. During the past week, how swollen was your lower limb? (Circle one response.)
  (1) Not at all
  (2) Mildly
  (3) Moderately
  (4) Very
  (5) Extremely.

During the past week, please tell us about how painful your lower limb was during the following activities. (Circle ONE response on each line that best describes your average ability.)

(3) Walking on flat surfaces?
  (1) Not painful
  (2) Mildly painful
  (3) Moderately painful
  (4) Very painful
  (5) Extremely painful
  (6) Could not do because of lower limb pain
  (7) Could not do for other reasons.
(4) Going up or down stairs?
  (1) Not painful
  (2) Mildly painful
  (3) Moderately painful
  (4) Very painful
  (5) Extremely painful
  (6) Could not do because of lower limb pain
  (7) Could not do for other reasons.
(5) Lying in bed at night?
  (1) Not painful
  (2) Mildly painful
  (3) Moderately painful
  (4) Very painful
  (5) Extremely painful
  (6) Could not do because of lower limb pain
  (7) Could not do for other reasons.
(6) Which of the following statements best describes your ability to get around most of the time during the past week? (Circle one response.)
  (1) I did not need support or assistance at all.
  (2) I mostly walked without support or assistance.
  (3) I mostly used one cane or crutch to help me get around.
  (4) I mostly used two canes, two crutches or a walker to help me get around.
  (5) I used a wheelchair.
  (6) I mostly used other supports or someone else had to help me get around.
  (7) I was unable to get around at all.
(7) How difficult was it for you to put on or take off socks/stockings during the past week? (Circle one response.)
  (1) Not at all difficult
  (2) A little bit difficult
  (3) Moderately difficult
  (4) Very difficult
  (5) Extremely difficult
  (6) Cannot do it at all.
